# Complex Regulatory Role of the TRPA1 Receptor in Acute and Chronic Airway Inflammation Mouse Models

**DOI:** 10.3390/ijms21114109

**Published:** 2020-06-09

**Authors:** Zsófia Hajna, Kata Csekő, Ágnes Kemény, László Kereskai, Tamás Kiss, Anikó Perkecz, István Szitter, Béla Kocsis, Erika Pintér, Zsuzsanna Helyes

**Affiliations:** 1 Department of Pharmacology and Pharmacotherapy, Medical School and János Szentágothai Research Centre & Centre for Neuroscience, University of Pécs, H-7624 Pécs, Hungary; zsofia.hajna@aok.pte.hu (Z.H.); kemenyagnes1@gmail.com (Á.K.); kiss891012@gmail.com (T.K.); perkecz@gmail.com (A.P.); szitteristvan@gmail.com (I.S.); erika.pinter@aok.pte.hu (E.P.); zsuzsanna.helyes@aok.pte.hu (Z.H.); 2 Department of Medical Biology, University of Pécs, Medical School, H-7624 Pécs, Hungary; 3 Department of Pathology, University of Pécs, Medical School, H-7624 Pécs, Hungary; kereskai.laszlo@pte.hu; 4 Department of Medical Microbiology and Immunology, University of Pécs, Medical School, H-7624 Pécs, Hungary; kocsis.bela@pte.hu; 5 PharmInVivo Ltd., H-7629 Pécs, Hungary

**Keywords:** bronchitis, cigarette smoke, COPD, emphysema, LPS, pneumonitis, whole body plethysmography

## Abstract

The Transient Receptor Potential Ankyrin 1 (TRPA1) cation channel expressed on capsaicin-sensitive afferents, immune and endothelial cells is activated by inflammatory mediators and exogenous irritants, e.g., endotoxins, nicotine, crotonaldehyde and acrolein. We investigated its involvement in acute and chronic pulmonary inflammation using *Trpa1* gene-deleted (*Trpa1^−/−^*) mice. Acute pneumonitis was evoked by intranasal *Escherichia coli* endotoxin (lipopolysaccharide: LPS) administration, chronic bronchitis by daily cigarette smoke exposure (CSE) for 4 months. Frequency, peak inspiratory/expiratory flows, minute ventilation determined by unrestrained whole-body plethysmography were significantly greater, while tidal volume, inspiratory/expiratory/relaxation times were smaller in *Trpa1^−/−^* mice. LPS-induced bronchial hyperreactivity, myeloperoxidase activity, frequency-decrease were significantly greater in *Trpa1^−/−^* mice. CSE significantly decreased tidal volume, minute ventilation, peak inspiratory/expiratory flows in wildtypes, but not in *Trpa1^−/−^* mice. CSE remarkably increased the mean linear intercept (histopathology), as an emphysema indicator after 2 months in wildtypes, but only after 4 months in *Trpa1^−/−^* mice. Semiquantitative histopathological scores were not different between strains in either models. TRPA1 has a complex role in basal airway function regulation and inflammatory mechanisms. It protects against LPS-induced acute pneumonitis and hyperresponsiveness, but is required for CSE-evoked emphysema and respiratory deterioration. Further research is needed to determine TRPA1 as a potential pharmacological target in the lung.

## 1. Introduction

The Transient Receptor Potential Ankyrin 1 (TRPA1) receptor is the sole member of the “ankyrin” subfamily of the Transient Receptor Potential (TRP) receptors in mammals. It is predominantly expressed on capsaicin-sensitive peptidergic sensory nerves densely innervating the lungs [1,2]. TRPA1 is a large transmembrane protein forming a ligand-gated non-selective cation channel [3,4].

TRPA1 is activated by several exogenous stimuli, such as bacterial endotoxin [5] and environmental irritants like acrolein, crotonaldehyde, nicotine and isocyanates [6,7,8,9,10], found in cigarette smoke, wood smoke, diesel exhaust and tear gas [6,7,10,11,12,13]. Moreover, several endogenous mediators produced during inflammation and oxidative stress, such as lipid peroxidation products, e.g., 4-hydroxynonenal (HNE) and 4-oxo-2-nonenal (4-ONE) [14,15,16], reactive oxygen species (ROS) [17], hydrogen sulfide, bradykinin and prostanoids stimulate TRPA1 [18,19,20,21,22]. Electrophilic compounds activate the receptor by covalently binding to the reactive cysteine residues at the intracellular N-terminal region [23,24,25,26]. Bradykinin and prostaglandins sensitize TRPA1 [22,27] through protein kinase A (PKA)-mediated phosphorylation [28,29]. Moreover, TRPA1 is also involved in protease activated receptor 1 (PAR1)/Gq-mediated increase of the intracellular Ca^2+^-level [30,31].

Pro-inflammatory sensory neuropeptides, e.g., substance P (SP) and calcitonin gene-related peptide (CGRP) are released in response to TRPA1 activation leading to neurogenic inflammation (vasodilatation and plasma protein extravasation) [32]. Simultaneously with these pro-inflammatory mediators, anti-inflammatory neuropeptides, e.g., somatostatin and pituitary adenylate cyclase activating polypeptide are also released from the same terminals counteracting the inflammatory process [33,34].

TRPA1 is also expressed on several non-neuronal cells [35], e.g., keratinocytes, macrophages and CD4+ lymphocytes [36,37,38], epidermal melanocytes, fibroblasts [39,40], urothelial and endothelial cells, as well as primary human osteoarthritic chondrocytes [41,42,43]. In the airways, TRPA1 is expressed on fibroblasts, tracheal, bronchial and alveolar epithelial cells, bronchial smooth muscle cells (SMC), as well as lymphocytes [17,44,45,46,47,48,49,50].

Due to its polymodal chemosensor function and its wide expression pattern, TRPA1 has been addressed as having a key role in physiological and pathophysiological processes, particularly in neuro-immune interactions [51,52,53]. It is suggested to be a particularly important chemical sensor in the respiratory system, playing a role in physiological (protective reflexes, cough and sneeze) and pathophysiological responses (inflammation, bronchial hyperreactivity) [54,55,56]. Although increasing evidence suggests TRPA1 involvement in the pathogenesis of chronic obstructive pulmonary disease (COPD), asthma, chronic cough, cystic fibrosis etc., pointing to the important therapeutic potential of TRPA1 in the pharmacological treatment of chronic pulmonary diseases [26,46,54,57,58,59,60,61,62], there are few in vivo data concerning its function in airway inflammation. Therefore, the results are far from being conclusive and more information is needed to determine the significance of TRPA1 as a possible pharmacological target in inflammatory lung disease, pneumonitis and COPD.

Therefore, in the present study we investigated the involvement of TRPA1 in the in vivo models of endotoxin (lipopolisaccharide: LPS)-induced acute and cigarette smoke exposure-induced chronic pulmonary inflammation with the help of TRPA1 wildtype (*Trpa1^+/+^*) and gene-deficient (*Trpa1^−/−^*) mice.

## 2. Results

### 2.1. Differences in Basal Airway Function Parameters of Trpa1^+/+^ and Trpa1^−/−^ Mice

**Figure 1:**
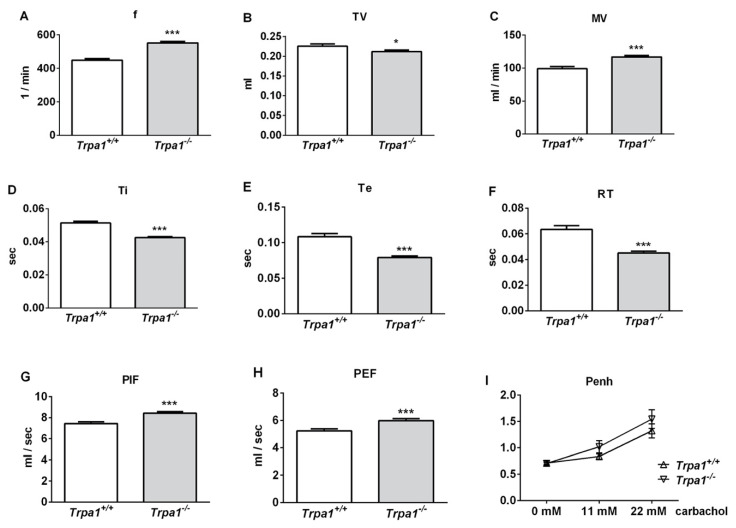
Comparison of the basal airway functions of intact *Trpa1^+/+^* and *Trpa1^−/−^* mice measured by unrestrained whole-body plethysmography. (**A**) Breathing frequency (f), (**B**) tidal volume (TV), (**C**) minute ventilation (MV), (**D**) inspiratory time (Ti), (**E**) expiratory time (Te), (**F**) relaxation time (RT), (**G**) peak inspiratory flow (PIF) and (**H**) peak expiratory flow (PEF) were measured in conscious, spontaneously breathing mice. (**I**) Enhanced pause (Penh) correlating with bronchial responsiveness was assessed by the nebulization of the muscarinic receptor agonist, carbachol. Values represent the means ± SEM, n = 30–35 mice/group; Student’s t-test for unpaired comparison; * *p* < 0.05; *** *p* < 0.001.

Under intact conditions, frequency (f), minute ventilation (MV), peak inspiratory flow (PIF) and peak expiratory flow (PEF) were significantly greater, tidal volume (TV), inspiratory time (Ti), expiratory time (Te) and relaxation time (RT) were significantly smaller, while no difference was detected in the enhanced pause (Penh) of *Trpa1^−/−^* mice compared to their wildtype counterparts, measured by unrestrained whole body plethysmography (WBP) (Figure 1).

### 2.2. Endotoxin-Induced Bronchial Hyperreactivity is Greater in Trpa1^−/−^ Mice

**Figure 2:**
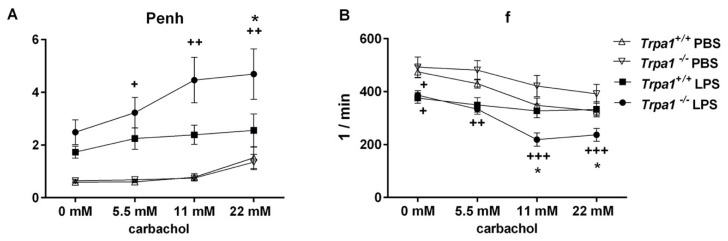
Inflammatory airway hyperreactivity: (**A**) carbachol-induced bronchoconstriction and (**B**) changes in breathing frequency. Carbachol-evoked bronchoconstriction remarkably increased, and the decrease in breathing frequency was significantly greater in *Trpa1^−/−^* mice 24 h after LPS administration, but not in the wildtypes. Values represent the means ± SEM, n = 5–10 mice/group; two-way ANOVA followed by Tukey’s post-test; ^+^ *p* < 0.05, ^++^ *p* < 0.005, ^+++^ *p* < 0.001 vs. PBS-treated respective group; * *p* < 0.05 vs. LPS-treated *Trpa1^+/+^.*

Bronchial responsiveness characterized by the Penh parameter significantly and concentration-dependently elevated in the *Trpa1^−/−^* mice in response to increasing concentrations of the muscarinic receptor agonist carbachol 24 h after the administration of intranasal LPS. This indicates the development of inflammatory airway hyperreactivity, which was significantly greater in the TRPA1-deficient mice compared to the LPS-treated wildtypes (Figure 2A).

LPS inhalation significantly reduced basal f in both strains. In response to carbachol, f did not change in *Trpa1^+/+^* mice, but it significantly declined in a concentration-dependent manner in the *Trpa1^−/−^* ones, being significantly lower compared to the wildtypes at 11 and 22 mM carbachol stimuli (Figure 2B).

Interestingly, the mild, although significant breathing frequency difference between the wildtype and knockout groups found under intact conditions was not observable in this model, most probably because of the different study setting and the use of phosphate buffer saline (PBS) in the control group.

### 2.3. Endotoxin-Induced Histological Alterations Show No Difference in the Case of TRPA1-Deficiency

**Figure 3:**
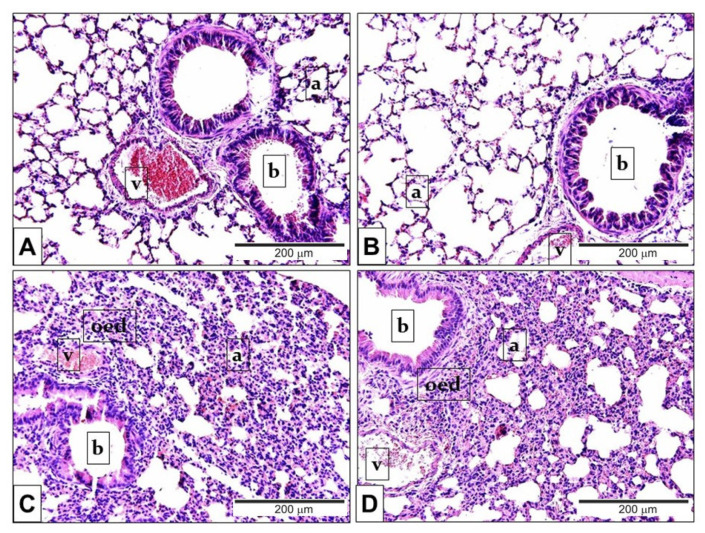
Representative histological pictures of LPS-induced pulmonary alterations. Compared to the PBS-treated (**A**) *Trpa1^+/+^* and (**B***) Trpa1^−/−^* control groups, the lung tissue of LPS-treated (**C**) *Trpa1^+/+^* and (**D**) *Trpa1^−/−^* mice exhibited remarkable perivascular and peribronchial oedema, neutrophil granulocyte accumulation, macrophage infiltration and goblet cell hyperplasia (hematoxylin-eosin staining; 200× *g* magnification; a: alveolus, b: bronchiolus, v: venula, oed: oedema).

**Figure 4:**
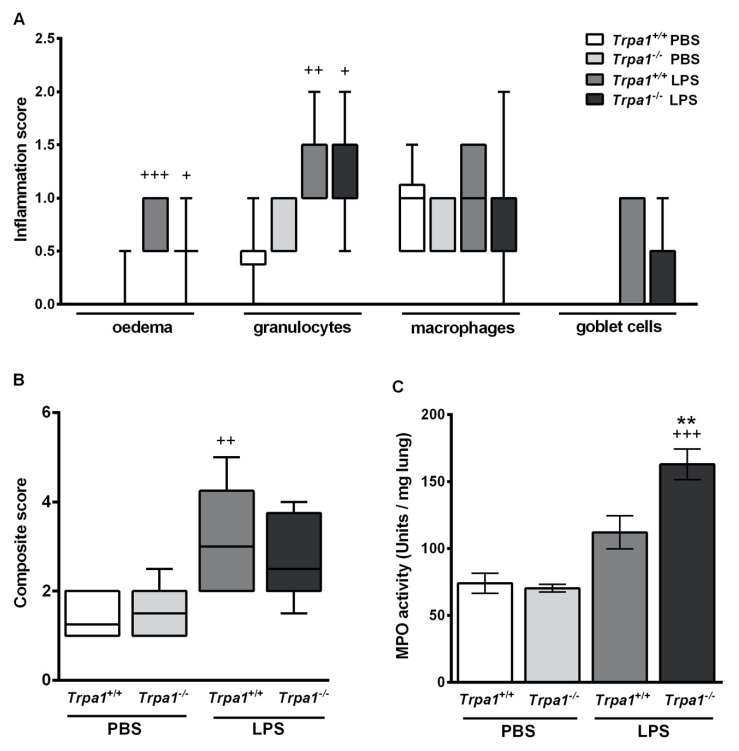
Semiquantitative histopathological evaluation and determination of the myeloperoxidase (MPO) activity of the lung 24 h after LPS inhalation. No significant difference was observed in the (**A**) detailed and (**B**) composite scores of histopathological inflammatory parameters between the *Trpa1^+/+^* and *Trpa1^−/−^* groups. Boxplots represent the minimum, first quartile, mean, third quartile and maximum values, n = 7–13/group, Kruskal–Wallis followed by Dunn’s post-test; ^+^ *p* < 0.05, ^++^ *p* < 0.005, ^+++^ *p* < 0.001 vs. PBS-treated *Trpa1^+/+^*. (**C**) LPS administration increased myeloperoxidase activity in the lung, which was significantly elevated in *Trpa1^−/−^* compared to the wildtypes. Values represent the means ± SEM, n = 5–8 mice/group; one-way ANOVA followed by Bonferroni’s post-test; ^+++^ *p* < 0.001 vs. PBS-treated respective group; ** *p* < 0.01 vs. LPS-treated *Trpa1^+/+^*.

In comparison to the PBS-treated mouse groups (Figure 3A,B) LPS administration induced remarkable oedema formation and inflammatory cell infiltration (Figure 3C,D) in the lung tissue. Based on the semiquantitative histopathological evaluation perivascular/peribronchial oedema and neutrophil granulocyte infiltration (Figure 4A) were significantly higher 24 h after LPS treatment, and the extent of goblet cell hyperplasia also increased, although not significantly. There was no statistical difference in the extent of endotoxin-induced airway inflammation scores between *Trpa1^+/+^* and *Trpa1^−/−^* mice (Figure 4A,B).

### 2.4. Endotoxin-Induced Myeloperoxidase Activity in the Lung is Greater in Trpa1^−/−^ Mice

Myeloperoxidase (MPO) enzyme activity correlating with the activated neutrophil granulocytes and macrophages increased 24 h after LPS-inhalation in both strains, however it was significantly greater in *Trpa1^−/−^* mice compared to the wildtypes (Figure 4C).

### 2.5. Chronic Cigarette Smoke Induces Respiratory Function Alterations in Trpa1^+/+^, but not in Trpa1^−/−^ Mice

**Figure 5:**
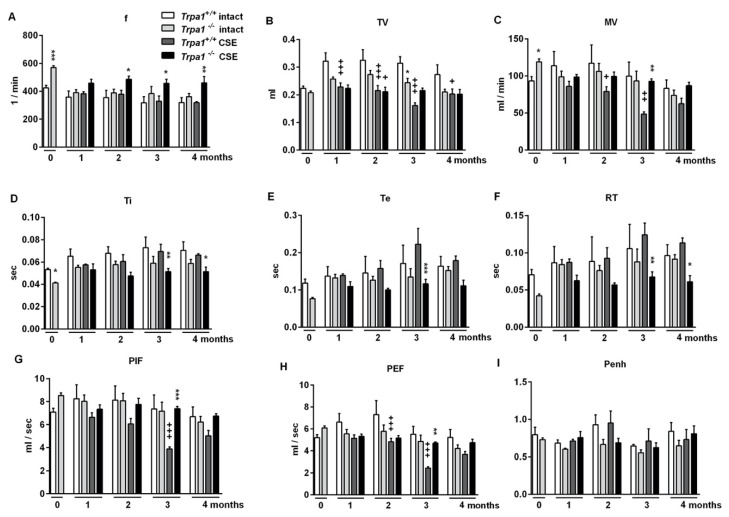
Respiratory functions during the 4 months of cigarette smoke exposure. (**B**) Tidal volume (TV), (**C**) minute ventilation (MV), (**G**) peak inspiratory flow (PIF) and (**H**) peak expiratory flow (PEF) significantly decreased after CSE with a peak at 3 months in *Trpa1^+/+^* mice. The significant strain differences in (**A**) frequency, (**D**) inspiratory time (Ti), (**E**) expiratory time (Te) and (**F**) relaxation time (RT) observed already in intact mice were not influenced by CSE. (**I**) Enhanced pause did not show any differences either by treatment or by strain. Values represent means ± SEM, n = 6–7 mice/group; two-way ANOVA followed by Tukey’s post-test; ^+^ *p* < 0.05, ^++^ *p* < 0.005, ^+++^ *p* < 0.001 vs. PBS-treated respective group; * *p* < 0.05, ** *p* < 0.005, *** *p* < 0.001 vs. LPS-treated *Trpa1^+/+^*.

Respiratory functions were measured in a follow-up design before and at the end of each month in the 4-month long protocol of cigarette smoke exposure (CSE). CSE induced a gradual and significant decrease in TV, MV, PIF and PEF in *Trpa1^+/+^* mice with a peak at 3 months, which was not present in the *Trpa1^−/−^* animals (Figure 5). The significant differences in f, Ti, Te and RT measured in the *Trpa1^−/−^* mice were attributable to the significant differences between the wildtype and gene-deficient mice observed already in the intact animals, before CSE.

### 2.6. CSE-Induced Inflammatory Histopathological Alterations are Similar in Trpa1^+/+^ and Trpa1^−/−^ Mice

**Figure 6:**
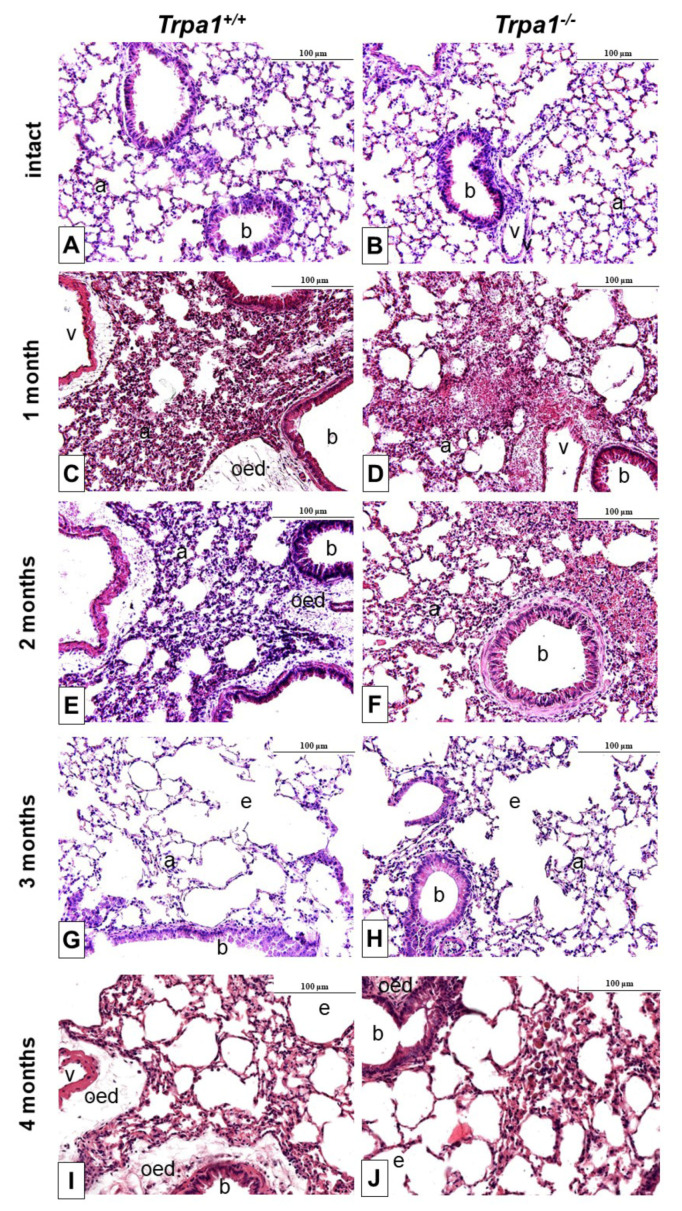
Representative histological pictures of the lungs of *Trpa1^+/+^* (**A**,**C**,**E**,**G**,**I**) and *Trpa1^−/−^* (**B**,**D**,**F**,**H**,**J**) mice under intact conditions (**A**,**B**), and after 1–4 months of CSE (**C**–**J**). One month of CSE induced a perivascular and peribronchial oedema formation associated with inflammatory cell infiltration, that gradually subsided over the 4-month long protocol. After the 3rd month of CSE, emphysema formation was observed in both wildtype and gene-deficient mice. (hematoxylin-eosin staining; 200× *g* magnification; a: alveolus, b: bronchiolus, v: venula, oed: oedema, e: emphysema).

**Figure 7:**
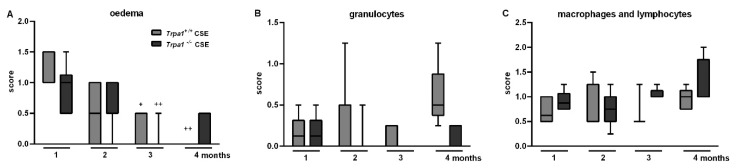
Semiquantitative evaluation of the histopathological changes of the lung after chronic cigarette smoke exposure. (**A**) Perivascular/peribronchial oedema developed after 1 month of CSE, which significantly decreased by time in both strains. The accumulation of perivascular and peribronchial, as well as interstitial (**B**) neutrophil granulocytes, (**C**) macrophages and lymphocytes did not differ in either groups significantly. Boxplots represent the minimum, first quartile, mean, third quartile and maximum values, n = 5–13/group, Kruskal–Wallis followed by Dunn’s post-test; ^+^ *p* < 0.05, ^++^ *p* < 0.005 vs. 1st month of CSE.

After one month of CSE remarkable perivascular oedema developed (Figure 6C,E) that significantly decreased by time in both strains, and almost completely resolved at the end of the 4th month (Figure 7A). However, the accumulation of perivascular and peribronchial, as well as interstitial neutrophil granulocytes, macrophages and lymphocytes remained moderately increased throughout the experimental protocol (Figure 7B,C). At the end of the 3rd month of CSE, structural destruction characteristic to emphysema (Figure 6G–J) already developed.

### 2.7. Cigarette Smoke-Induced Emphysema Develops Earlier in Trpa1^+/+^ Mice

**Figure 8:**
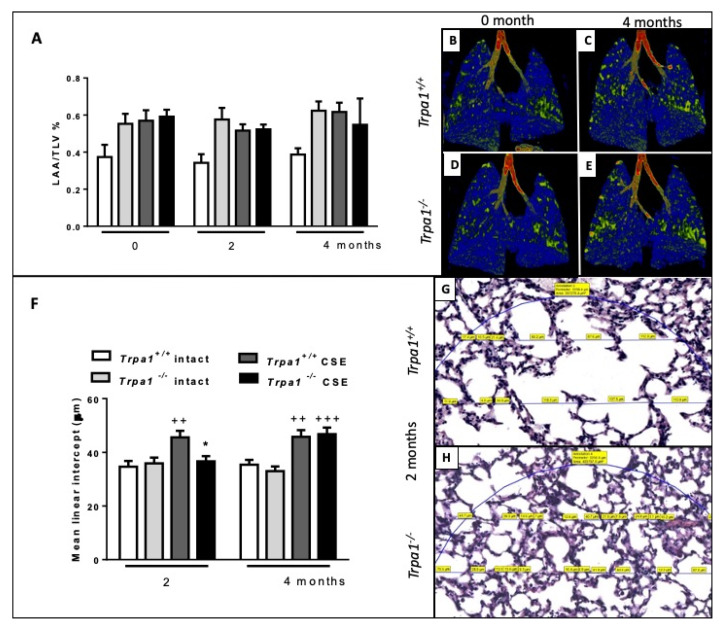
Quantitative evaluation of emphysema. (**A**) LAA/TLV ratio correlating with air-filled regions of the lungs exposed to CSE showed no significant alterations. Representative microCT pictures of lungs (**B**,**D**) before and (**C**,**E**) after 4 months of CSE in *Trpa1^+/+^* and *Trpa1^−/−^* mice, respectively. (**F**) Microscopic quantitative assessment of Lm showed a significant increase in *Trpa1^+/+^* already after 2 months of CSE, but not in the *Trpa1^−/−^*. Values represent means ± SEM, n = 6–7 mice/group; repeated measures two-way ANOVA followed by Tukey’s post-test. Representative microscopic pictures of (**G**) *Trpa1^+/+^* and (**H**) *Trpa1^−/−^* mouse lung tissues after 2 months of CSE. Values represent means ± SEM, n = 60–100 measurements/group; two-way ANOVA followed by Tukey’s post-test; ^++^ *p* < 0.005, ^+++^ *p* < 0.001 vs. PBS-treated respective group; * *p* < 0.05 vs. CSE-treated *Trpa1^+/+^.* (The blue circle indicates the region of interest in which Lm was measured. The measured chord lengths represented by blue lines were expressed in µm on the scanned slides and labelled in yellow.).

Emphysema was quantified by in vivo microCT by the ratio of the low attenuation area (LAA) and total lung volume (TLV) before and after 2 and 4 months of CSE. The ratio correlating with the extent of air-filled regions did not show alterations either by CSE treatment or time (Figure 8A). However, the more sensitive mean linear intercept (Lm) measurement revealed that in *Trpa1^+/+^* mice emphysema already started to develop at an earlier timepoint compared to the gene-deleted counterparts, Lm was significantly increased in the wildtypes after 2 months of CSE, however, not in the *Trpa1^−/−^*. At the end of the 4th month, Lm was elevated in both strains exposed to CS (Figure 8F).

### 2.8. Chronic Cigarette Smoke-Induced Inflammatory Cell Accumulation in the BALF Shows No Strain Difference

**Figure 9:**
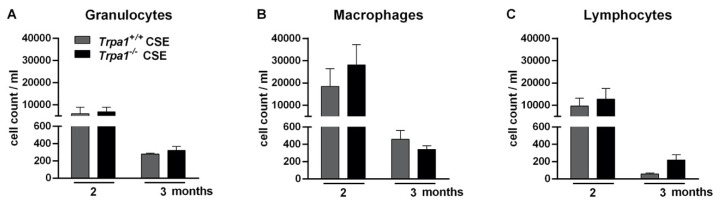
Number of inflammatory cells in the bronchoalveolar lavage fluid (BALF) after 2–3 months of CSE in *Trpa1*^+/+^ and *Trpa1*^−/−^ mice. After the peak of inflammatory cell infiltration at a 2-month timepoint, the number of (**A**) granulocytes, (**B**) macrophages and (**C**) lymphocytes remarkably decreased by the end of the 3rd month in both *Trpa1^+/+^* and *Trpa1^−/−^* mice. Values represent means ± SEM, n = 6–7 mice/group; two-way ANOVA followed by Tukey’s post-test.

CSE induced massive accumulations of granulocytes, macrophages and lymphocytes measured in the bronchoalveolar lavage fluid (BALF) of both *Trpa1^+/+^* and *Trpa1^−/−^* mice. In agreement with our previous findings [63], the number of inflammatory cells reduced by the end of the 3rd month of CSE. There was no biologically relevant difference between the wildtype and gene-deficient mice in either inflammatory cell component (Figure 9).

## 3. Discussion and Conclusion

We provide here the first evidence that the TRPA1 channel has a complex role in basal airway function regulation and inflammatory mechanisms. It protects against LPS-induced acute pneumonitis and hyperresponsiveness, but triggers chronic CSE-evoked emphysema formation and respiratory deterioration (Table 1).

Increasing data have suggested the involvement of the TRPA1 receptor in the pathophysiological mechanisms of several airway diseases, such as asthma, COPD and allergic/irritative cough [7,46,56,57,58,59,60,62,64,65,66,67], but little is known about its activation mechanisms and its potential as a drug target in lung inflammation is still controversial. Our results demonstrating seemingly opposing regulatory functions of TRPA1 in the acute and chronic models can be explained by its multiple localizations that might evoke different responses. Neuronal TRPA1 in the airways is expressed almost exclusively on vagal afferents that are a subpopulation of sensory afferents. It is co-localized with Transient Receptor Potential Vanilloid 1 (TRPV1) on capsaicin-sensitive vagal bronchopulmonary nociceptive C-fibers originating from the jugular ganglion. Most of these peptidergic bronchopulmonary C-fibers modulate the inflammatory process and induce defensive reflexes, shallow breathing, mucus secretion [68,69]. However, TRPA1-mediated effects via the sensory trigeminal afferentation of the upper airways also might contribute to our results in both models [70]. Besides the neuronal expression, TRPA1 is also located on epithelial, inflammatory and bronchial smooth muscle cells [6,47,48,50].

The virtually contradicting role of TRPA1 might also be explained by the very distinct pathophysiological mechanisms of the LPS and CSE models involving different signaling pathways in different cell types. Moreover, endotoxin and cigarette smoke components are able to directly activate TRPA1 among other exogenous irritants reaching the airways, besides a variety of endogenous inflammatory mediators (protons, hydrogen peroxide, prostaglandins etc.) [5,8,10,18,22].

Direct interactions between endotoxins and TRPA1 [71,72] has been described in LPS-induced acute inflammatory pain, CGRP-release and vascular response that developed via the activation of TRPA1 on nociceptive sensory fibers, independently from TLR4 signaling [5]. The TRPA1 agonist cinnamaldehyde exerts complex immunomodulatory effects on LPS-induced systemic inflammatory response syndrome [73]. TRPA1 mediates the LPS-induced inflammatory responses in primary human osteoarthritic fibroblast-like synoviocytes [74]. The role of TRPA1 as an LPS sensor is suggested to be evolutionarily conserved [75], that occurs via mechanical modifications of the plasma lipid membrane. Interestingly, *Escherichia coli* (*E. coli*) LPS seems to be more effective on TRPA1 than other types of endotoxins [76]. Although previous studies demonstrate TRPA1 activation by LPS and its implication in acute LPS-induced pain and neurogenic inflammation [5], the relative importance of TLR4 and TRPA1 activation by LPS in our pneumonitis model remains to be elucidated.

The protective role of TRPA1 in the LPS-induced acute inflammatory processes including bronchial hyperreactivity is supported by previous results of our group reporting similar protective actions of capsaicin-sensitive sensory nerves and the TRPV1 receptor in the same model [77,78]. The similar functions of TRPA1 and TRPV1 ion channels are not surprising, since they are often co-expressed and interact with each other on the peptidergic sensory nerves [1,79]. The TRPA1-mediated protective effect might be explained by the release of anti-inflammatory sensory neuropeptides from primary afferents, such as somatostatin, similarly to the mechanism described earlier for TRPV1 activation [34,77].

TRPA1 proved to be protective against inflammatory bronchial hyperresponsiveness potentially via smooth muscle relaxant neuropeptides released from the sensory nerves, despite its presence on bronchial smooth muscle cells [47], which might directly lead to bronchoconstriction. Furthermore, bronchodilator effects of TRPA1 activation via non-epithelial PGE_2_ production was recently reported in allergic airway inflammation models [80]. Such mechanism might also be involved in our results. A recent study also demonstrated significantly increased airway hyperresponsiveness in *Trpv4* and *Trpa1/Trpv1* double knockout mice as well. The TRPV4 antagonist HC067047 administered to *Trpa1/Trpv1* double knockout animals further increased the Penh value [81] supporting the protective role of these TRP channels in the LPS-induced acute airway inflammation model.

TRPA1-deficiency did not significantly alter inflammatory cell numbers in the histopathological pictures, but significantly increased neutrophil- and macrophage-derived MPO activity, which is supported by TRPA1 expression on these cells [36,82]. The discrepancies between the histopathological semiquantitative scores and the MPO activity might be due to the different sensitivity of the functional and morphological assessments, however, it has also been described that MPO protein expression itself might not correlate strongly with MPO activity due to genetic polymorphism or endogenous inhibitors [83]. Consequently, increased ROS production might contribute to the increased bronchial hyperreactivity in the *Trpa1*-deleted mice. These results are supported by earlier data that TRPA1 activation by irritants evokes defensive functions in the airways [54].

Despite these protective functions of TRPA1 in the acute LPS-evoked pneumonitis model, TRPA1 activation is required for chronic CSE-evoked emphysema and respiratory deterioration, such as MV, TV, PIF and PEF decrease with a peak after 3 months.

These data are supported by findings, that 4-week CSE increases TRPA1 mRNA level in the nodose and jugular ganglia, positively correlating with the inflammatory cell infiltration in the BALF [84]. Moreover, cigarette smoke extract also increased the expression of TRPA1 in airway epithelial cells in a hypoxia-inducible factor-1α-mediated manner [17,85]. Mostly in vitro data using cigarette smoke extract are available about the role of TRPA1 in cigarette smoke-induced airway inflammation. Isolated bronchi experiments suggested that cigarette smoke extract, as well as its components (acrolein and crotonaldehyde) caused Ca^2+^-dependent CGRP- and SP-release from the capsaicin-sensitive nerve endings, and TRPA1 was involved in the cigarette smoke extract-induced tracheal plasma extravasation and bronchoconstriction [6]. Cigarette smoke-induced CGRP release in the trachea were predominantly mediated by TRPA1 rather than nicotinic receptors [9]. Cigarette smoke and acrolein released keratinocyte chemoattractant (CXCL1/KC, mouse analogue of IL-8), which was attenuated by TRPA1 antagonists and TRPA1-deficiency [47]. Furthermore, cigarette smoke extract-induced and TRPA1-mediated IL-8 release was shown to develop via NADPH-oxidase activation and the MAPKs/NFκB signaling pathway-related Ca^2+^ influx [17]. TRPA1 is involved in cigarette smoke extract-evoked alveolar and bronchial epithelial damage [49]. Nicotine directly activates the TRPA1 receptor [8,10], which might mediate bronchoconstriction [47]. Similarly, ROS and several lipid peroxidation products also stimulate TRPA1, which is likely to contribute to oxidative stress-evoked airway pathologies induced by CSE [7,17,55], such as emphysema, for which we provided the first data here.

The extent of perivascular/peribronchial oedema was the most severe after 1 month and gradually decreasing afterwards, and the inflammatory cell infiltration reached its maximum after 2 months of CSE in both groups, which is in agreement with our earlier findings in the same model [63]. TRPA1 deficiency did not result in significant changes of the cellular components of these chronic inflammatory processes, as shown by both the histopathological results and BALF analysis.

Since the activation of TRPA1 exerts protective effects in LPS-induced acute pneumonitis and subsequent bronchial hyperreactivity, our findings clearly support the concept that short-term activation of TRPA1 results in defensive effects presumably via sensory nerves and consequently released protective neuropeptides. However, permanent stimulation of the receptor under chronic inflammatory conditions of the airways results in complex regulatory functions due to the diverse localization of TRPA1 also on non-neural cells and its broad range of both exogenous and endogenous activators.

The genetic deletion of the receptor does not directly predict prophylactic or therapeutic potential of TRPA1 agonists or antagonists. However, the activation of TRPA1 by commonly inhaled substances, e.g., cinnamaldehyde or carvacrol [7] (components of cinnamon or thyme essential oils) could be beneficial against acute inflammatory changes of the lung, additionally considering their antimicrobial potentials against pathogens predominant in airway infections [86].

Therefore, further research is needed to determine TRPA1 potential as a pharmacological target in the lung.

## 4. Methods

### 4.1. Animals

Experiments were performed on male and female *Trpa1^−/−^* mice and their *Trpa1^+/+^* counterparts (8–10 weeks, 20–25 g). The original heterozygote *Trpa1*^+/−^ breeding pairs were a generous gift of Pierangelo Geppetti (Firenze, Italy) [87]. The endotoxin-induced pneumonitis model was carried out on female animals, while in the cigarette smoke-induced bronchitis model mice of both sexes were used. Background strain of the gene-deleted animals was C57Bl/6, and the germline transmission of the mutated allele and excision of the selection cassette were verified by PCR analysis. Animals were bred and kept in the Laboratory Animal House of the Department of Pharmacology and Pharmacotherapy, University of Pécs, Pécs, Hungary at 24–25 °C, provided with standard chow and water *ad libitum* and maintained under a 12-h light–dark cycle.

### 4.2. Ethics

All experimental procedures were carried out according to the 40/2013 (II.14.) Government Regulation on Animal Protection, Consideration Decree of Scientific Procedures of Animal Experiments (243/1988) and Directive 2010/63/EU of the European Parliament. Studies were approved and a license was given by the Ethics Committee on Animal Research of University of Pécs, Pécs, Hungary according to the Ethical Codex of Animal Experiments (licence No.: BA02/2000-35/2016, BA02/2000-26/2018). We addressed the ARRIVE guidelines for designing, performing and reporting the experiments wherever possible.

### 4.3. Endotoxin-Induced Acute Pneumonitis

Acute interstitial lung inflammation was evoked by intranasal (i.n.) administration of 60 μL, 167 µg/mL *E. coli* (serotype: O83) endotoxin (lipopolysaccharide, LPS) under light ether anaesthesia [77]. LPS was isolated and purified in the Department of Microbiology, University of Pécs, Pécs, Hungary [88]. Control animals received the same volume of sterile PBS.

Endotoxin is a component of the cell wall of Gram-negative bacteria, composed of a phosphoglycolipid (lipid A) covalently bound to a hydrophilic heteropolysaccharide part [89]. LPS—connected to the specific LPS binding protein (LBP)—is able to bind to Toll-like receptor 4 (TLR4)-CD14 glycoprotein complex expressed on macrophages and monocytes [90]. Via the p38-MAPK signaling pathway it induces the activation and accumulation of immune cells [91,92]. As a result, inflammatory cytokines and proinflammatory mediators (e.g., bradykinin, leukotrienes and prostaglandins) are released from the activated immune cells directly activating the sensory nerve endings [93]. Beside the activation of immune cells, LPS also elicits bronchial contraction in the lung causing enhanced airway resistance [94].

### 4.4. Cigarette Smoke-Induced Chronic Airway Inflammation

Chronic bronchitis was elicited by whole body cigarette smoke exposure for 4 months. Mice were exposed to cigarette smoke (3R4F Kentucky Research Cigarette; University of Kentucky, Lexington, KY, USA) twice daily, 10 times per week in a whole body smoke exposure chamber (Teague Enterprises, Woodland, CA, USA) for 30 min followed by a ventilation period of 30 min as described previously [63].

### 4.5. Investigation of Airway Function and Bronchial Responsiveness

Parameters of the airway function were measured in conscious, spontaneously breathing mice with the help of unrestrained whole-body plethysmography (Buxco Europe Ltd., Winchester, UK).

In the endotoxin-induced acute pneumonitis model, breathing frequency (f) and carbachol-induced airway reactivity were measured before and 24 h after intranasal PBS/LPS administration [95]. Bronchoconstriction was induced by increasing concentrations (5.5, 11 and 22 mM) of the muscarinic receptor agonist carbachol (50 µL/mouse), and the Penh (enhanced pause) value was determined. Penh is a complex calculated parameter ((expiratory time/relaxation time) − 1)/(max. expiratory flow/max. inspiratory flow), well correlating with bronchoconstriction and airway resistance measured in ventilated animals using invasive techniques [34].

Considering that cigarette smoke-induced chronic bronchitis model is a disease model with high translational relevance, we performed an extended analysis of the respiratory functions determining f, TV, MV, Ti, Te, RT, PIF, PEF and Penh before and at the end of every month of the 4-month-investigational period as described previously [63].

### 4.6. Cell Composition Analysis of the Bronchoalveolar Lavage Fluid (BALF)

In the cigarette smoke exposure model, mice were anaesthetised with ketamine and xylazine (100 mg/kg and 5 mg/kg, s.c., respectively) and the lungs were washed with 5 mL PBS. BALF was collected in centrifuge tubes (1000 rpm, 5 min) and the supernatants were removed. Cells were resuspended in staining buffer (0.1% NaN_3_ and 0.1% BSA dissolved in 500 µL PBS), and 1 µL CD45 fluorescein-5-isothiocyanate (FITC) solution was given to the samples. After incubation of 30 min, 1 µL propidium-iodide was given to the samples and after centrifugation (1000 rpm, 5 min) the supernatant was removed again. Cells were resuspended in fixation buffer (3% formaldehyde dissolved in 700 µL PBS) and the cell profile of the samples was analysed with a CyFlow Space flow cytometer (Sysmex Partec, Münster, Germany) [96].

We previously showed that in the CSE model perivascular/peribronchial oedema developed in the 1st month, while in the 2nd and 3rd months inflammatory cell infiltration dominated the histopathological picture, which decreased by the 4th month accompanied by tissue destruction [63]. Based on these data, we investigated the amount of inflammatory cells in the BALF after the 2nd and 3rd months.

### 4.7. Histological Examination of the Lungs

In both models, lungs were removed following cervical dislocation in deep anaesthesia. The left lung was fixed in 4% formaldehyde and embedded in paraffin for histopathological processing. Sections were made with 5–7 µm microtome and stained with hematoxylin-eosin (HE). One section of the lungs of the endotoxin-treated mice was stained with periodic acid-Schiff (PAS) reagent in order to visualize mucus producing goblet cells. Semiquantitative evaluation of the histological alterations was performed by an expert pathologist blinded from the experimental design.

In the acute pneumonitis model, semiquantitative histopathological scoring of the lungs was performed based on the following parameters: perivascular oedema (0–3), accumulation of the perivascular and peribonchial neutrophil granulocytes (0–3), infiltration of activated macrophages and mononuclear cells into the alveolar space (0–2), as well as hyperplasia of the bronchiolar goblet cells (0–2) [97]. The composite inflammatory score (ranging between 0 and 10) was generated by addition of the individual histopathological parameters.

In the chronic COPD model, semiquantitative histopathological scoring of the lungs was established by our work group and performed on the basis of perivascular oedema, the accumulation of neutrophil granulocytes into the perivascular/peribronchial and interstitial space, as well as the perivascular/peribronchial and interstitial infiltration of macrophages and lymphocytes with scores ranging from 0–3 (0: intact, 0.5: focal mild, 1: diffuse mild, 1.5: focal moderate, 2: diffuse moderate, 2.5: focal severe, 3: diffuse severe).

Quantitative histopathological evaluation of emphysema was performed by the measurement of the mean linear intercept as described previously [98]. Briefly, air space enlargement was assessed by the measurement of the alveolar space or alveolar and ductal air space along parallel lines on approximately 400,000 µm^2^ representative areas on each slide by The Case Viewer software (3DHISTECH, Budapest, Hungary).

### 4.8. Morphological Evaluation of the Lungs with In Vivo MicroCT Investigation

In the cigarette smoke exposure model, structural alterations, such as the LAA/TLV% characteristic to emphysema was also measured by in vivo microCT in a self-controlled manner, before, as well as 2 and 4 months after CSE exposure as described previously [63]. Briefly, mice were anaesthetized with pentobarbital (70 mg/kg) i.p. and their lungs were imaged by breath-gated tomography on a Skyscan 1176 high resolution microtomograph (Skyscan, Kontich, Belgium). Reconstruction and morphometric analysis were performed with the software provided by the manufacturer.

### 4.9. Determination of Myeloperoxidase (MPO) Activity from Lung Homogenates with Spectrophotometry

During the evolvement of endotoxin-induced acute pneumonitis, MPO enzyme is produced by the accumulated neutrophil granulocytes, monocytes and macrophages [99]. The MPO activity of the lung homogenates were measured by spectrophotometry using H_2_O_2_-3,3′,5,5′-tetramethyl-benzidine (TMB/H_2_O_2_) and compared to a standard MPO preparation as described earlier [98].

### 4.10. Statistical Analysis

Statistical analysis was performed by the GraphPad Prism v6 software (GraphPad Software, San Diego, CA, USA). Comparison of basal respiratory functions of intact wildtype and TRPA-deficient mice was performed by a Student’s t-test for unpaired comparison. Parameters of the respiratory function in both the LPS and CSE study, as well as LAA/TLV%, mean linear intercept, and BALF inflammatory cell counts were evaluated by two-way ANOVA followed by Tukey’s post hoc test. MPO activity was analysed by one-way ANOVA followed by Bonferroni’s post-test. Histopathological semiquantitative scores were evaluated by Kruskal–Wallis analysis followed by Dunn’s post-test. In all cases *p* < 0.05 was accepted as significant.

## Figures and Tables

**Table 1:** The complex regulatory role of TRPA1 in acute and chronic airway inflammation (ns: non-significant; arrows indicate change compared to control PBS-treated or non-inflamed intact mice, ^++^ *p* < 0.005, ^+++^ *p* < 0.001; asterisks indicate strain difference, * *p* < 0.05, ** *p* < 0.005 vs. *Trpa1^+/+^*).

**Endotoxin-Induced Acute Inflammation**	***Trpa1^+/+^***	***Trpa1^−/−^***	***Trpa1^+/+^* vs. *Trpa1^−/−^*** ***LPS-Treated Inflamed***
	**Compared to PBS-Treated Control**		
**Bronchial hyperreactivity**	**ns.**	↑(++)	*
Myeloperoxidase activity	ns.	↑(+++)	**
Inflammatory histopathological changes	↑(++)	ns	ns
**CSE-induced Chronic Inflammation**	***Trpa1^+/+^***	***Trpa1^−/−^***	***Trpa1^+/+^* vs. *Trpa1^−/−^*** ***CSE-Treated Inflamed***
	**Compared to Intact Control**		
TV, MV, PIF, PEF	↓(++/+++)	ns	*
Oedema	↑	↑	ns.
Inflammatory cell infiltration	↑	↑	ns.
Emphysema	earlier	later	*
